# Dietary Intake of Flavonoids and Ventilatory Function in European Adults: A GA^2^LEN Study

**DOI:** 10.3390/nu10010095

**Published:** 2018-01-15

**Authors:** Vanessa Garcia-Larsen, Narjis Thawer, David Charles, Aedin Cassidy, Thibaut van Zele, Trine Thilsing, Matti Ahlström, Tari Haahtela, Thomas Keil, Paolo M Matricardi, Grzegorz Brożek, Marek L Kowalski, Joanna Makowska, Ewa Niżankowska-Mogilnicka, Barbara Rymarczyk, Carlos Loureiro, Ana Todo Bom, Claus Bachert, Bertil Forsberg, Christer Janson, Kjell Torén, James F Potts, Peter GJ Burney

**Affiliations:** 1 Program in Human Nutrition, Department of International Health, Johns Hopkins Bloomberg School of Public Health, Baltimore, MD 21205, USA; 2 Respiratory Epidemiology and Public Health Group, National Heart and Lung Institute, Imperial College London, London SW7 1BU, UK; narjis.thawer@gmail.com (N.T.); d.charles@smd15.qmul.ac.uk (D.C.); j.potts@imperial.ac.uk (J.F.P.); p.burney@imperial.ac.uk (P.G.J.B.); 3 Barts and the London School of Medicine, Queen Mary University of London, London E1 1BZ, UK; 4 Department of Nutrition, Norwich Medical School, University of East Anglia, Norwich NR4 7TJ, UK; a.cassidy@uea.ac.uk; 5 Upper Airway Research Laboratory, Ghent University, 9000 Ghent, Belgium; thibaut.vanzele@ugent.be; 6 Research Unit of General Practice, Department of Public Health, University of Southern Denmark, 5000 Odense C; Denmark; tthilsing@health.sdu.dk; 7 Skin and Allergy Hospital, Helsinki University Hospital, 00029 HUS Helsinki, Finland; matti.ahlstrom@helsinki.fi (M.A.); tari.haahtela@hus.fi (T.H.); 8 Deptartment of Pediatrics, Charité-Universitätsmedizin Berlin, 10117 Berlin, Germany; thomas.keil@charite.de; 9 Institute of Social Medicine, Epidemiology and Health Economics, Charité-Universitätsmedizin, 10117 Berlin, Germany; 10 Institute of Clinical Epidemiology and Biometry, Würzburg University, 97070 Würzburg, Germany; paolo.matricardi@charite.de; 11 Department of Epidemiology, College of Medicine, Medical University of Silesia, 40-752 Katowice, Poland; brozekg@mp.pl; 12 Department of Immunology, Rheumatology and Allergy, Medical University of Lodz, 90-647 Lodz, Poland; kowalsml@csk.umed.lodz.pl (M.L.K.); joanna.makowska@umed.lodz.pl (J.M.); 13 Jagiellonian University School of Medicine, 31-008 Krakow, Poland; ewan@ghml.pl; 14 Clinical Department of Internal Diseases, Allergology and Clinical Immunology, Medical University of Silesia, 40-055 Katowice, Poland; b.rymarczyk@interia.pl; 15 Department of Immuno-Allergology, Coimbra University Hospital, 3000-075 Coimbra, Portugal; acnl@sapo.pt (C.L.); flcosta@netcabo.pt (A.T.B.); 16 Division of ENT Diseases, Karolinska Institute, 171 77 Stockholm, Sweden; claus.bachert@ugent.be; 17 Division of Occupational and Environmental Medicine, Department of Public Health and Clinical Medicine, Umeå University, 901 87 Umeå, Sweden; bertil.forsberg@envmed.umu.se; 18 Department of Medical Sciences, Respiratory, Allergy and Sleep Research, Uppsala University, 751 85 Uppsala, Sweden; christer.janson@medsci.uu.se; 19 Section of Occupational and Environmental Medicine, University of Gothenburg, 405 30 Gothenburg, Sweden; kjell.toren@amm.gu.se

**Keywords:** flavonoids, pro-anthocyanidins, lung function, GA^2^LEN

## Abstract

Background: Flavonoids exert anti-inflammatory properties and modulate oxidative stress in vitro, suggesting a protective effect on lung function, but epidemiological studies examining this association are scarce. Methods: A stratified random sample was drawn from the GA^2^LEN screening survey, in which 55,000 adults aged 15 to 75 answered a questionnaire on respiratory symptoms. Post-bronchodilator spirometry was obtained from 2850 subjects. Forced vital capacity (FVC), the ratio between the forced exhaled volume in 1 second (FEV_1_) and FVC (FEV_1_/FVC), FVC below lower limit of normal (FVC < LLN), and FEV_1_/FVC < LLN were calculated. Intake of the six main subclasses of flavonoids was estimated using the GA^2^LEN Food Frequency Questionnaire. Adjusted associations between outcomes and each subclass of flavonoids were examined with multivariate regressions. Simes’ procedure was used to test for multiple comparisons. Results: A total of 2599 subjects had valid lung function and dietary data. A lower prevalence of FVC < LLN (airway restriction) was observed in those with higher total flavonoid (adjusted odds ratio (aOR), higher vs. lowest quintile intake 0.58; 95% Confidence Interval (CI) 0.36, 0.94), and pro-anthocyanidin intakes (aOR 0.47; 95% CI 0.27, 0.81). A higher FEV_1_/FVC was associated with higher intakes of total flavonoids and pro-anthocyanidins (adjusted correlation coefficient (a β-coeff 0.33; 0.10, 0.57 and a β-coeff 0.44; 95% CI 0.19, 0.69, respectively). After Simes’ procedure, the statistical significance of each of these associations was attenuated but remained below 0.05, with the exception of total flavonoids and airway restriction. Conclusions: This population-based study in European adults provides cross-sectional evidence of a positive association of total flavonoid intake and pro-anthocyanidins and ventilatory function, and a negative association with spirometric restriction in European adults.

## 1. Introduction

Flavonoids are polyphenolic plant secondary metabolites ubiquitously present in vegetables, fruits, and beverages. Various biological properties of relevance for lung health have been identified for these compounds, including antioxidant, anti-allergic, and immune-modulating activities [1]. There is a considerable body of experimental evidence (in vivo and in vitro) on the anti-inflammatory properties of flavonoids, particularly pro-anthocyanidins, on lung health. Experimental evidence shows that pro-anthocyanidins can reduce lung mRNA expression of pro-inflammatory cytokines such as Interferon-γ, Interleukin (IL)-4, and IL-13 [2], and down-regulate nitric oxide production in asthma [3]. Trifolirhizin, another flavonoid, has been demonstrated to inhibit in a dose-response manner, the production of IL-6 and tumour necrosis factor-α (TNF-α) in lung cancer cells [4]. Pro-anthocyanin-enriched blackcurrant extract and epigallocatechins were shown to reduce eosinophilic-driven airway inflammation in human alveolar cells [5].

Epidemiological studies have shown a beneficial effect of a higher intake of fruits and vegetables on chronic respiratory health in adults, most of which refers to incidence of asthma and chronic obstructive pulmonary disease (COPD) [6,7] or to prevalence of asthma symptoms [8]. These benefits have been attributed to the high content of flavonoids present in hard fruits such as apples [9,10], though this effect was not confirmed in one population-based study [11]. Epidemiological evidence on the association between flavonoids and lung function is scant and limited to the assessment of few flavonoid subclasses (e.g., flavonols and flavan-3-ols). A recent population-based study in young adults found that a higher forced vital capacity (FVC) was associated with a higher intake of catechins and of total fruit [12]. There are also observational studies showing a slower decline of forced exhale volume in 1 second (FEV_1_) [13,14] and FVC [14], indirectly attributed to flavonoids. In asthmatic subjects, a higher FEV_1_ was associated with having a higher intake of soy genistein (a type of polyphenol) [15].

The GA^2^LEN survey is the first multi-national, population-based study investigating the role of all main major subclasses of flavonoids on ventilatory function in a representative sample of European adults.

## 2. Materials and Methods

### 2.1. The GA^2^LEN Study: Screening and Clinical Surveys

The core protocol for the GA^2^LEN survey required 18 European participating centres to identify a random sample of at least 3000 adults aged 15–74 years from an available population-based sampling frame. In 2008–2009, potential participants were sent a short questionnaire by mail, and at least three attempts were made to elicit a response. The questionnaire collected information on age, gender, smoking, and the presence of symptoms of asthma (including age of onset), chronic rhino-sinusitis, and allergic rhinitis. Four sub-samples were selected to define cases and controls: (1) those with self-reported asthma and at least one respiratory symptom reported in the last 12 months (‘asthma’); (2) those having chronic sinusitis (defined following the European Position Paper on Rhinosinusitis and Nasal Polyps (EP^3^OS) Group criteria, that is, the presence of at least two of the following symptoms for at least 12 weeks in the past year: (i) nasal blockage, (ii) nasal discharge, (iii) facial pain or pressure, or (iv) reduction in sense of smell with at least one of the symptoms being nasal blockage or nasal discharge); (3) those who had both ‘asthma’ and ‘chronic sinusitis’; and (4) those who had none of these conditions [16].

Participants selected were invited in for further tests. Relevant to this analysis they had performed spirometry using a New Diagnostic Design (NDD) Easy-One spirometer before and after 200 µg of short-acting beta-agonist, salbutamol inspired through a Clement Clarke Able spacer. After at least three acceptable and two reproducible maneuvers were obtained by trained technicians, the lung function was defined as the highest of two values for FVC and FEV_1_ taken from acceptable forced expiratory maneuvers that did not vary by more than 200 mL. Only spirometry that met the European Respiratory Society/American Thoracic Society (ERS/ATS) criteria were accepted [17]. All spirometries were checked centrally for quality control.

### 2.2. Estimates of Diet and Flavonoid Intake

Usual consumption of 250 foods was estimated using the GA^2^LEN food frequency questionnaire (FFQ), which was designed to assess dietary intake across countries in Europe using a single, standardised instrument, and validated in five countries participating in GA^2^LEN ClinicalTrials.gov Identifier: NCT03251157) [18]. The FFQ collected data on a wide range of foods, including 37 vegetables and 28 fruits, as well as foods rich in soy and grains. The FFQ also included a general question to ascertain use of nutritional supplements (‘Do you regularly take any nutritional supplements?’).

On the FFQs, respondents sometimes left individual items blank. This was assumed to denote zero intake of these foods; however, if 20% of the items were blank, the FFQ was considered incomplete, and the subject was excluded from further analyses. Participants were also excluded if they had extreme values of total energy intake, which might suggest an unrealistic response. We calculated the expected basal metabolic rate (BMR) with the given age, weight, and sex, and excluded subjects with a ratio of energy intake to expected BMR that was either below the 0.05th sample centile or above the 99.5th sample centile for their country.

Total energy intake (TEI) was calculated using the latest available food composition estimates from the British Food Composition Table [19]. A database for the assessment of intakes of different flavonoid subclasses was constructed. We used the updated and expanded US Department of Agriculture (USDA) flavonoid content of foods and the pro-anthocyanidin databases [20,21]. Intakes were derived for the six main flavonoid subclasses habitually consumed in general population [22]: (1) flavanones (eriodictyol, hesperetin, and naringenin); (2) anthocyanins (cyanidin, delphinidin, malvidin, pelargonidin, petunidin, and peonidin); (3) flavan-3-ols (catechins and epicatachins); (4) flavonols (quercetin, kaempferol, myricetin, and isohamnetin); (5) flavones (luteolin and apigenin); and (6) polymers (proanthocyanidins, theaflavins, and thearubigins). Total flavonoid intakes were derived by the addition of the six component subclasses. We also examined associations with the pro-anthocyanidins subclass, which were derived separately, summing monomers, dimers, trimers, 4- to 6-mers, 7- to 10-mers, and >10-mers.

### 2.3. Statistical Analyses

Sampling probability weights were used to standardise prevalences by gender and age to a European Standard Population. For each centre, the sample mean of the FEV_1_/FVC ratio and the FVC was estimated as well as the sample prevalence of a low FEV_1_/FVC ratio or a low FVC according to the National Health and Nutrition Examination Survey (NHANES) III norms for Caucasians. For each flavonoid group, total intake was split into quintiles. For each centre, the odds of the FEV_1_/FVC ratio being below the lower limit of normal comparing the lowest and highest quintile of flavonoid consumption was calculated. These odds ratios for all centres were then combined into a single estimate using random effects meta-analysis [23]. For continuous measures of lung function (FEV_1_/FVC and FVC), their association with each flavonoid subclass was assessed against the per-quintile increase of each flavonoid subclass. Associations were examined using two models of adjustments. Model 1 included adjustment for height, age, and sex, and Model 2 added the variables whether a case or a control (as defined in the GA^2^LEN survey), body mass index (BMI), smoking status (never, ex-smoker, current smoker), occupation, age at completion of full time education, use of nutritional supplements, total fruit and vegetable intake, and TEI. Simes’ procedure was used to correct statistical estimates derived from multiple testing [24].

We examined the correlation of each of the subclasses of flavonoids and these ranged between 0.28 (flavan-3-ols) and 0.40 (pro-anthocyanidins), therefore we did not use energy-adjusted values of flavonoids and instead adjusted for TEI in the models.

Written informed consent was obtained from all participants. The GA^2^LEN Follow-Up Survey was granted ethical approval by the UK’s National Ethics Research Committee No. 07/H0604/121.

## 3. Results

A total of 2599 adults (mean age 47.2 ± 14.5 years) had valid lung function estimates (Table 1). With the exception of Belgians, the BMI for all participant countries indicated that the participants were, on average, overweight. Around half of the participants reported never having smoked, whilst just over 15% declared that they were current smokers. Nearly two-thirds of the sample studied was defined as a ‘case’. Use of nutritional supplements varied across countries, with Finnish adults reporting the highest proportion of intakes of nutritional supplements and Portuguese the lowest. Intake of fruits and vegetables averaged three portions of each per person per day in the whole sample.

**Figure 1:**
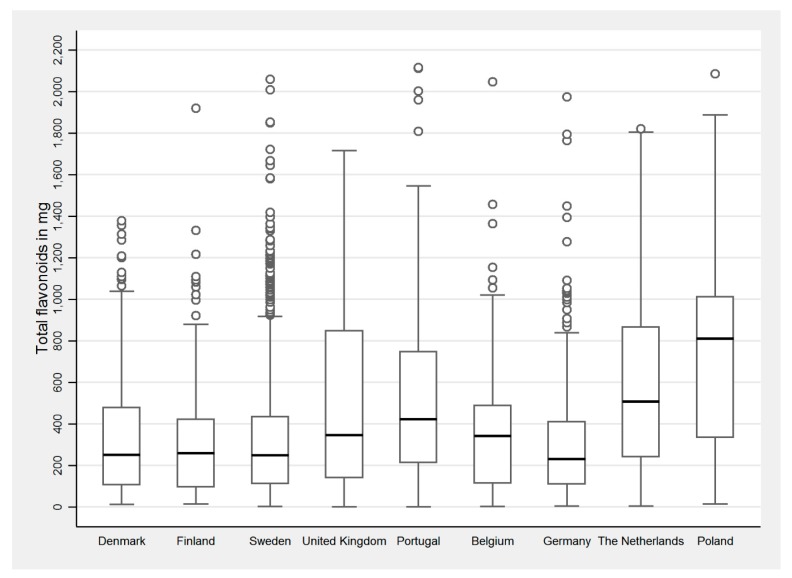
Total flavonoid intake in the adults participating in the GA^2^LEN Follow-Up Survey who had valid lung function data (*n* = 2599).

The distribution of total flavonoid intake is shown in Figure 1, and that of other subclasses in Table 2. Overall, the median intake of total flavonoids was 291 mg/person/day. Germany and Belgium had the lowest intake. Poland, the Netherlands, and Portugal had the highest intakes of total flavonoids. The Scandinavian countries and the UK had an intermediate intake. The main contributors to total flavonoid intake are summarised in Table 3. Fruits were the main source, followed by tea, wine, chocolate, and vegetables. As expected, flavonoid polymers were the main source of flavonoids in most food groups. Citrus fruits were the predominant source of flavonones. Fruits (mostly berries and citrus) and wine were the main contributors of anthocyanins, whilst flavonols and flavones were mainly derived from vegetables (Table 3).

The association between FVC and spirometric restriction (FVC < LLN) with flavonoids is presented in Table 4. In the basic model (adjusted by height, age, and sex only), total flavonoid intake and several most subclasses were positively associated with a 30 mL higher FVC. After controlling for further confounders (BMI, whether a case or a control, intake of nutrient supplements, education, smoking status, total fruit and vegetable intake, and TEI), these associations were attenuated and were no longer statistically significant. With regards to spirometric restriction, a 42% lower risk of FVC < LLN was observed in those with the highest quintile of total flavonoid intake (odds ratio (OR) 0.58; 95% CI 0.36, 0.94). Similarly, after adjusting for the full set of potential confounders, a 53% lower risk of FVC < LLN was observed in those adults with the highest intake of anthocyanins and pro-anthocyanidins. After applying the Simes’ procedure, the statistical significance of the adjusted associations remained (Table 4).

Table 5 shows the association between FEV_1_/FVC and FEV_1_/FVC < LLN with flavonoid intake. A higher FEV_1_/FVC was associated with a higher intake of flavonoids, polymers, and pro-anthocyanidins. After Simes’ procedure, each of these associations remained statistically significant. Airflow obstruction (FEV_1_/FVC < LLN) was not associated with any of the flavonoid exposures studied.

**Figure 2:**
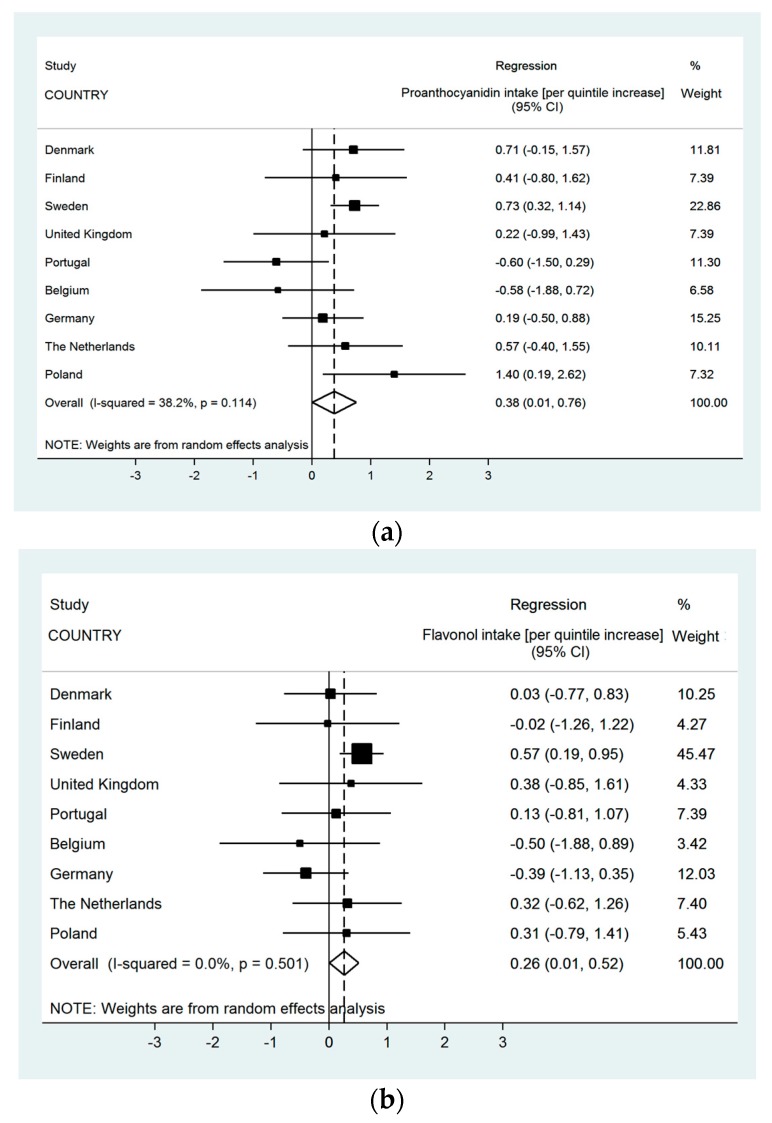
The association of FEV1/FVC and intakes (mg) of pro-anthocyanidins (**a**) and flavonols (**b**) (Meta-analysis of adjusted effect estimates (β-coefficient); *n* = 2599).

The meta-analyses in Figure 2 illustrate the adjusted associations between FEV_1_/FVC and total flavonols and pro-anthocyanidins per centre and the overall effect. These associations showed evidence of moderate but not statistically significant evidence of heterogeneity between countries in the analyses with pro-anthocyanidins (*I*^2^ = 38.2%, *p*-value = 0.10), whilst the meta-analysis for pro-anthocyanidins showed no evidence of variations between countries, as reflected by the zero heterogeneity in these associations.

## 4. Discussion

This multi-centric population-based study in European adults showed that those with a higher intake of pro-anthocyanidins, had a higher FEV_1_/FVC, a lower risk of airway obstruction (FEV_1_/FVC < LLN) and a lower risk of spirometric restriction (FVC < LLN). Total flavonoid intake was also associated with a higher FEV_1_/FVC and with a lower risk of spirometric restriction. The statistical significance of each of these associations remained after applying Simes’ procedure, with the exception of the associations observed with FEV_1_/FVC < LLN. The direction of these associations was fairly consistent across countries, with moderate or no evidence of heterogeneity between them.

To our knowledge, this is the first multi-national population-based study to examine the association between objective measures of airway restriction and obstruction with the intake of a wide range of flavonoids. Our results suggest that a higher intake of flavonoids might contribute to a better ventilatory function and reduce the risk of spirometric restriction. Two other observational studies have suggested this, although they examined three subclasses of flavonoids, and the effect of pro-anthocyanidins was not investigated. The study of Garcia-Larsen recently showed that catechin intake was associated with a 70 mL higher FVC in a population-based study of young adults, when comparing those in the highest versus the lowest quintile of intake [14]. Similarly, Tabak et al. showed that total intake of catechins was associated with a 43 mL higher FEV_1_ and negatively associated with self-reported symptoms in Dutch adults [25].

After controlling for multiple comparisons, our results showed no evidence of an association with airflow obstruction, but with spirometric restriction. FVC has been linked more strongly to survival and to comorbidities than airflow obstruction [26]. This remains largely unexplained, other than a strong relation to early life factors such as birth weight. The fact that a beneficial association was observed with FVC < LLN and not with measures of airway obstruction, suggest that pro-anthocyanidins might contribute to preserving ventilatory function and might, in turn, prevent an accelerated decline of FVC, thus reducing the risk of general mortality and co-morbidities [27]. Although such an effect would need to be confirmed in longitudinal studies, the findings in the GA^2^LEN population justify the need for such studies.

Mechanistic effects from experimental studies support our findings. An experimental study using a mouse model to investigate the effect of pro-anthocyanidins on lung inflammation showed that the animals fed with this flavonoid had a significantly reduced production of pro-inflammatory cytokines produced by Th2 cells [2]. The antioxidant activity of pro-anthocyanidins has also been demonstrated in murine model of induced allergic asthma [3,26], where mice treated with the flavonoid extract had significantly lower nitric oxide levels [3], airway hyper-responsiveness [26], and their lung tissue levels of various cytokines, chemokines, and growth factors were lower than those in the mice that did not receive the pro-anthocyanidin extract [27]. In human alveolar cells, pro-anthocyanidin extract has been shown to reduce the IL-4-dependent eosinophil expression [5].

Earlier evidence in humans and rodents showing that flavonoids are poorly absorbed and often excreted un-metabolised [28], has been reappraised in the light of more recent studies. The study of Czek and colleagues showed that specific flavonoids such as anthocyanidins are metabolised to a structurally diverse range of metabolites [29], and that these circulate in plasma in adults for up to 48 hours after ingestion [30]. In animals fed with procyanidin-enriched supplement, metabolites of pro-anthocyanidins (mainly glucoronide forms of epicatechins) were detected in plasma and also found in lung tissue, up to 18 h after ingestion [31]. Metabolites of anthocyanins have also been detected after experimental ingestion in animals [32,33]. These findings suggest that the bioavailability of flavonoids expands beyond the gastro-intestinal tract and that they might play a key role in a number of anti-inflammatory processes in the airways [34].

In the GA^2^LEN study, we observed that with the exception of Germany, there was a consistent trend across countries for a reduced risk of spirometric restriction with higher in those with the highest intakes of pro-anthocyanidins, with no statistically significant evidence of heterogeneity. The risk reduction of 57% has clinical and public health relevance. The intake of pro-anthocyanidins in the GA^2^LEN participants was on average 192 mg and came largely from fruit intake. This amount can be achieved by eating 3–4 units of hard fruits a day, which is an attainable target in the general adult population. The statistical significance of this association was retained after applying Simes’ procedure. Although the statistical significance of the positive association between FVC and intake of pro-anthocyanidins was attenuated after Simes’ procedure, the size of the effect, and the consistent trend across countries found in this study, would confirm the laboratory evidence showing a protective effect of these specific flavonoids.

The associations between flavonoid subclasses and respiratory outcomes were controlled for use of nutritional supplements. Nearly a third of the participants in this study reported taking nutritional supplements, a strong trend widespread in Europe [35]. Bioactive forms of various polyphenols, including flavonoids, are often present in nutritional supplements, and our results are unlikely to be explained by this use.

The strengths of the current study include that we used a representative sample of the European adult population and used several measures of airway restriction and airway obstruction that underwent rigorous quality control before being considered acceptable. We also assessed a wide range of flavonoid subclasses. Dietary flavonoid intakes were calculated from a database developed using recent USDA databases for flavonoids and pro-anthocyanidins [21,22]. These datasets allowed us to quantify a broad range of flavonoid subclass intakes providing more robust evidence than previous studies on flavonoid intake and lung diseases. The FFQ used in the current study included a comprehensive list of fruits and vegetables, as well as other dietary sources known to have flavonoids. The FFQ was validated in a sample of this study population [17] and reflects the usual dietary intake of these individuals as well as facilitating international comparisons. We also validated the FFQ for intake of flavonoids and found that the FFQ is an accurate instrument to capture dietary sources of flavonoids [36].

This study has some limitations. High flavonoid intake may be representative of a healthy lifestyle in general, and although we adjusted for a range of lifestyle variables, as well as fruit and vegetable intake, residual or unmeasured confounding cannot be ruled out. The cross-sectional nature of our study prevents us from establishing a causal inference in the association between flavonoids and lung function. However, recent longitudinal findings from the elderly adults from the Normative Aging Study showed that a higher intake of anthocyanins was associated with slower FVC decline over 15 years [37], which lends further support to our hypothesis that a higher intake of flavonoids might preserve lung function and possibly reduce the risk of COPD. Epidemiological studies on lung health and flavonoids are still scant, with the exception of lung cancer, for which there is increasing evidence of a protective role of dietary flavonoids [38]. Replications of our findings in longitudinal studies are warranted to confirm the role in reducing the risk of lung disease.

## 5. Conclusions

In conclusion, this study provides for the first time, observational evidence for a positive association of pro-anthocyanidins and possibly total flavonoid intake and lung function in a multi-centric sample of European adults. The magnitude of the effect is clinically significant and of public health relevance. Increasing the consumption of dietary sources of flavonoids is an achievable target and can contribute to maintaining lung function, and possibly to preventing its accelerated decline.

## Figures and Tables

**Table 1:** General characteristics of the study population (*n* = 2599) ^#^.

**Variables**	**Countries Participating in the GA^2^LEN Nutrition Survey**									
	**Denmark**		**Finland**		**Sweden**		**UK**		**Portugal**	
	**Odense (268)**		**Helsinki (122)**		**Total (1085)**		**Total (139)**		**Coimbra (233)**	
Age, years; mean (SD)	47.9	(14.2)	45.8	(14.5)	45.5	(15)	51.3	(13.2)	47.2	(14.5)
Males, *n* (%)	115	(42.9)	47	(38.5)	471	(43.4)	54	(38.8)	80	(34.3)
Height, m (SD)	1.7	(0.1)	1.7	(0.1)	1.7	(0.1)	1.7	(0.1)	1.6	(0.1)
BMI (m^2^/kg)	26.9	(4.7)	26.5	(4.3)	26	(4.7)	27.8	(5.8)	26.6	(5.3)
Never smokers, n (%)	119	(44.4)	60	(49.2)	578	(53.3)	63	(45.3)	148	(63.5)
Ex-smokers, n (%)	71	(26.5)	30	(24.6)	366	(33.7)	55	(39.6)	51	(21.9)
Current smokers, n (%)	78	(29.1)	32	(26.2)	141	(13)	21	(15.1)	34	(14.6)
Cases/controls	180/88		62/60		774/311		95/44		169/64	
* FVC, L	4.1	(1.0)	4.0	(1.0)	4.1	(1.0)	3.7	(1.0)	3.8	(1.0)
* FEV_1_/FVC	78.4	(8.6)	80	(7.9)	78.2	(8.6)	75.4	(10.8)	80.5	(8.3)
FVC < LLN, n (%)	36	(13.4)	10	(8.2)	141	(13)	20	(14.4)	16	(6.9)
FEV_1_/FVC < LLN, n (%)	32	(11.9)	6	(4.9)	130	(12)	25	(18)	25	(10.7)
Nutritional supplements, n (%)	107	(39.9)	52	(42.6)	285	(26.3)	49	(35.3)	15	(6.4)
Total fruit intake, g/day	328	(263.8)	278.2	(219.4)	320.7	(242.5)	328.8	(238.3)	549.2	(403.7)
Total vegetable intake, g/day	342.6	(223.8)	415.5	(297.5)	355.8	(206.8)	343.2	(207.8)	454.5	(321.1)
**Variables**	**Countries Participating in the GA^2^LEN Nutrition Survey**									
	**Belgium**		**Germany**		**The Netherlands**		**Poland**		**Total**	
	**Ghent (107)**		**Total (305)**		**Amsterdam (174)**		**Total (166)**		**2599**	
Age, years, mean (SD)	44.5	(14.4)	48.7	(15.2)	52.7	(13.6)	51.6	(15)	47.4	(14.8)
Males, n (%)	47	(43.9)	116	(38)	88	(50.6)	65	(39.2)	1083	(41.7)
Height, m (SD)	1.7	(0.1)	1.7	(0.1)	1.7	(0.1)	1.7	(0.1)	1.7	(0.1)
BMI (m^2^/kg)	24.5	(4)	26.9	(5)	27.1	(4)	27.7	(5.2)	26.5	(4.8)
Never smokers, n (%)	60	(56.1)	148	(48.5)	70	(40.2)	79	(47.6)	1,325	(51)
Ex-smokers, n (%)	32	(29.9)	108	(35.4)	67	(38.5)	48	(28.9)	828	(31.9)
Current smokers, n (%)	15	(14)	49	(16.1)	37	(21.3)	39	(23.5)	446	(17.2)
Cases/Controls	60/47		142/163		111/63		108/58		1701/898	
* FVC, L	4.3	(1.0)	3.9	(1.0)	4.1	(1.0)	3.8	(1.0)	4.0	(1.0)
* FEV_1_/FVC	79.4	(9.5)	78.8	(7.7)	77.1	(8.4)	77	(10.4)	78.3	(8.8)
FVC < LLN, n (%)	9	(8.4)	55	(18)	11	(6.3)	13	(7.8)	311	(12)
FEV_1_/FVC < LLN, n (%)	10	(9.3)	25	(8.2)	20	(11.5)	20	(12)	293	(11.3)
Nutritional supplements, n (%)	33	(30.8)	81	(26.6)	72	(41.4)	39	(23.5)	733	(28.2)
Total fruit intake, g/day	333.1	(235.1)	314.7	(273.2)	289.5	(211.8)	465.2	(466.9)	347.3	(291.2)
Total vegetable intake, g/day	244.8	(126.1)	261.9	(187.2)	224.5	(130.5)	482.8	(429.6)	349.1	(246.9)

FVC, forced vital capacity; FEV_1_, forced exhaled volume in 1 second; LLN, lower limit of normal. * Post-bronchodilator; SD standard deviation; ‘n’ number of participants; ‘%’ percentage; ^#^ Based on individuals with complete dietary data and valid (quality controlled) lung function measurements.

**Table 2:** Dietary intake of main flavonoid subclasses in adults from countries participating in GA^2^LEN.

	**Dietary Intake of Flavonoids across Countries (Median Intake in mg, IQR)**									
**Flavonoid Subclass**	**Denmark**		**Finland**		**Sweden**		**UK**		**Portugal**	
	**Odense (268)**		**Helsinki (122)**		**Total (1085)**		**Total (139)**		**Coimbra (233)**	
Total flavonoids	251.1	(106.3 to 479.7)	260.5	(95.2 to 427.2)	249.4	(113 to 439.1)	345.5	(140.3 to 848.8)	426.5	(215.8 to 749.1)
Flavanones	4.5	(1.3 to 20.9)	8.3	(1.8 to 23.6)	5.3	(1.4 to 21)	5	(1.8 to 18.3)	17.1	(2.9 to 60.3)
Anthocyanins	7.5	(2.4 to 16.3)	5.9	(2.1 to 13)	6.5	(2.1 to 15.1)	9.8	(3.7 to 30.3)	22.1	(6.9 to 47.5)
Flavan-3-ols	30.6	(14 to 69)	24.4	(10 to 56.3)	29.4	(13 to 65.3)	43.7	(14.8 to 183.5)	44	(20.9 to 78.2)
Flavanols	16.3	(9.3 to 29.3)	19.5	(10.8 to 33.1)	16.9	(9.2 to 28.2)	16.7	(9.1 to 32.7)	26.6	(12.9 to 44.6)
Flavones	2.1	(0.9 to 4.4)	2.4	(1.3 to 4.5)	2	(0.9 to 4.3)	1.7	(0.6 to 3.2)	2.6	(1.1 to 5.7)
Polymers	175.7	(66.1 to 316.3)	162.9	(62.5 to 260.2)	159.5	(66.4 to 283.3)	251.5	(75.7 to 601.5)	266.8	(106.5 to 459.8)
Pro-anthocyanidins	135.3	(68.6 to 261.1)	152.8	(58.7 to 270.7)	126.6	(62.9 to 245.7)	195.2	(76.7 to 376.6)	286.7	(113.1 to 465.1)
	**Dietary Intake of Flavonoids across Countries (Median Intake in mg, IQR)**									
**Flavonoid Subclass**	**Belgium**		**Germany**		**The Netherlands**		**Poland**		**Total**	
	**Ghent (107)**		**Total (305)**		**Amsterdam (174)**		**Total (166)**		**2599**	
Total flavonoids	341.4	(113.9 to 489.4)	231.7	(110.2 to 413.8)	507	(242.1 to 867.4)	817.3	(341.6 to 1029.3)	291.2	(126.8 to 569.4)
Flavanones	10.3	(2.3 to 27.9)	4.9	(1.1 to 21.6)	7	(2.3 to 29)	6.7	(1.8 to 28)	5.7	(1.5 to 22.5)
Anthocyanins	10.5	(2.7 to 21)	5.5	(1.7 to 13.3)	8.1	(2.8 to 22.1)	9.2	(2.3 to 26.3)	7.5	(2.4 to 19.6)
Flavan-3-ols	32.9	(13.6 to 83.8)	38.1	(13.2 to 77.3)	84.1	(35.5 to 186.2)	176.3	(54.9 to 200.1)	37.9	(15.4 to 90.4)
Flavanols	13.1	(7 to 23.7)	11	(5.2 to 20.2)	17.8	(9.2 to 30.6)	34	(21.3 to 51.8)	17.2	(9.2 to 30.8)
Flavones	1.4	(0.5 to 2.5)	1.1	(0.4 to 2.3)	1.5	(0.6 to 2.5)	2.1	(0.7 to 5.8)	1.9	(0.7 to 3.9)
Polymers	216.5	(71.1 to 325.5)	132.1	(63.7 to 268.1)	341.7	(129.5 to 622.3)	585.9	(243 to 687.5)	198.4	(72.9 to 375.2)
Pro-anthocyanidins	214.9	(80.5 to 343.8)	136.1	(69 to 261.4)	190.8	(101.5 to 327.8)	192.7	(105.5 to 311.8)	154.6	(72.5 to 284.3)

IQR: interquartile range.

**Table 3:** Provenance and contribution of flavonoid subclasses in the diet of participants in the GA^2^LEN Follow-Up Study.

	Food Groups (mg/% Total Dietary Sources)										
Flavonoid Subclasses (mg)	All Fruit	Citrus Fruit	Hard Fruit	Berries	Other Fruit	Vegetables	Chocolate	Nuts	Legumes	Tea & Coffee	Wine & Beer
Total flavonoids	289.93	36.58	124.08	2.91	64.09	14.9	30.87	1.43	0.23	152.9	21.07
Flavanones	7.51%	59.21%	0.00%	0.10%	0.19%	2.08%	0.00%	0.06%	0.00%	0.00%	2.47%
Anthocyanins	5.66%	9.00%	4.38%	28.94%	13.18%	1.96%	0.00%	2.59%	0.42%	0.00%	9.43%
Flavan-3-ols	6.86%	1.58%	7.07%	4.61%	8.93%	0.00%	16.33%	3.26%	21.38%	31.41%	26.85%
Flavonols	3.45%	1.49%	4.41%	1.17%	1.87%	79.15%	0.00%	1.38%	21.62%	2.91%	9.00%
Flavones	0.34%	1.62%	0.14%	0.45%	0.29%	16.81%	0.00%	0.00%	0.00%	0.00%	1.23%
Polymers	76.18%	27.11%	84.02%	64.74%	75.54%	0.00%	83.67%	92.71%	56.59%	65.68%	51.02%
Pro-anthocyanidins	234.07	10.11	110.84	1.98	50.75	0.00	34.64	1.40	0.18	15.1	14.92

**Table 4:** Association of post-bronchodilator spirometric values of FVC and FVC < LLN with dietary intake of flavonoids.

Flavonoid Subclass (Quintiles; mg)	FVC (Lt) (Continuous)						FVC < LLN (Binary)						
	Effect Size (Regression Coefficient and 95% Confidence Interval) Per-Quintile Increase in Flavonoid Intake						OR (95% Confidence Interval) Highest Vs. Lowest Quintile of Flavonoid Intake						
	Model 1 (*n* = 2599)		*p*-Value	Model 2 (*n* = 2599)		*p*-Value	Model 1 (*n* = 2599)		*p*-Value	Model 2 (*n* = 2599)		*p*-Value	*p*-Value after Simes’ Procedure
Total flavanoids	0.03	(0.02 to 0.05)	<0.0001	0.02	(−0.002 to 0.03)	0.09	0.47	(0.31 to 0.71)	<0.0001	0.58	(0.36 to 0.94)	0.03	0.07
Flavanones	0.03	(0.02 to 0.05)	<0.0001	0.02	(−0.002 to 0.03)	0.08	0.47	(0.31 to 0.71)	0.0003	0.60	(0.37 to 0.97)	0.04	0.07
Anthocyanins	0.04	(0.02 to 0.05)	<0.0001	0.02	(−0.01 to 0.04)	0.14	0.37	(0.24 to 0.57)	<0.0001	0.47	(0.26 to 0.83)	0.01	0.04
Flavan-3-ols	0.02	(0.002 to 0.03)	0.03	<0.001	(−0.02 to 0.02)	0.97	0.58	(0.39 to 0.86)	0.01	0.75	(0.49 to 1.14)	0.17	
Flavonols	0.03	(0.02 to 0.05)	0.0001	0.01	(−0.004 to 0.03)	0.12	0.52	(0.35 to 0.76)	0.0008	0.66	(0.41 to 1.04)	0.07	
Flavones	0.02	(0.01 to 0.04)	0.003	0.01	(−0.01 to 0.02)	0.54	0.58	(0.40 to 0.85)	0.01	0.79	(0.51 to 1.22)	0.28	
Polymers	0.03	(0.02 to 0.05)	0.0001	0.01	(−0.003 to 0.03)	0.10	0.52	(0.34 to 0.79)	0.002	0.65	(0.41 to 1.04)	0.07	
Pro-anthocyanidins	0.04	(0.02 to 0.05)	<0.0001	0.02	(−0.002 to 0.04)	0.07	0.41	(0.26 to 0.63)	0.0001	0.47	(0.27 to 0.81)	0.01	0.04

Model 1 Adjusted by height, age, and sex; Model 2 Adjusted by height, age, sex, whether a case or a control, BMI, smoking status (never, ex-smoker, current smoker), occupation, age at completion of full time education, use of nutritional supplements, total fruit intake, and total energy intake (TEI), Lt: liters.

**Table 5:** Association of post-bronchodilator ratio FEV_1_/FVC and FEV_1_/FVC < LLN with dietary intake of flavonoids.

Flavonoid Subclass (Quintiles; mg)	FEV_1_/FVC (Continuous)							FEV_1_/FVC < LLN (Binary)						
	Effect Size (Regression Coefficient and 95% Confidence Interval) Per-Quintile Increase in Flavonoid Intake							OR (95% Confidence Interval) Highest vs. Lowest Quintile of Flavonoid Intake						
	Model 1 (*n* = 2599)		*p*-Value	Model 2 (*n* = 2599)		*p*-Value	*p*-Value after Simes’ Procedure	Model 1 (*n* = 2599)		*p*-Value	Model 2 (*n* = 2599)		*p*-Value	*p*-Value after Simes’ Procedure
Total flavanoids	0.50	(0.29 to 0.72)	<0.0001	0.33	(0.10 to 0.57)	0.01	0.02	0.58	(0.39 to 0.86)	0.01	0.61	(0.39 to 0.97)	0.04	0.12
Flavanones	0.16	(−0.05 to 0.37)	0.15	0.07	(−0.30 to 0.17)	0.57		0.71	(0.47 to 1.08)	0.11	0.84	(0.51 to 1.37)	0.49	
Anthocyanins	0.34	(0.13 to 0.56)	0.002	0.12	(−0.15 to 0.39)	0.39		0.58	(0.39 to 0.86)	0.01	0.73	(0.43 to 1.24)	0.25	
Flavan-3-ols	0.42	(0.21 to 0.63)	0.0001	0.20	(−0.02 to 0.42)	0.07		0.65	(0.44 to 0.97)	0.03	0.73	(0.47 to 1.11)	0.14	
Flavonols	0.34	(0.13 to 0.56)	0.002	0.18	(−0.06 to 0.42)	0.14		0.77	(0.52 to 1.14)	0.19	0.92	(0.58 to 1.47)	0.74	
Flavones	0.31	(0.1 to 0.52)	0.004	0.17	(−0.07 to 0.4)	0.16		0.80	(0.54 to 1.18)	0.26	1.01	(0.63 to 1.6)	0.97	
Polymers	0.48	(0.27 to 0.69)	<0.0001	0.32	(0.09 to 0.55)	0.01	0.02	0.55	(0.37 to 0.81)	0.002	0.56	(0.36 to 0.88)	0.01	0.09
Pro-anthocyanidins	0.58	(0.37 to 0.79)	<0.0001	0.44	(0.19 to 0.69)	0.001	0.004	0.58	(0.39 to 0.85)	0.01	0.61	(0.37 to 0.99)	0.04	0.12
